# Arginine Metabolism in Bacterial Pathogenesis and Cancer Therapy

**DOI:** 10.3390/ijms17030363

**Published:** 2016-03-11

**Authors:** Lifeng Xiong, Jade L. L. Teng, Michael G. Botelho, Regina C. Lo, Susanna K. P. Lau, Patrick C. Y. Woo

**Affiliations:** 1 Department of Microbiology, The University of Hong Kong, Hong Kong, China; lfxiong@hku.hk (L.X.); llteng@hku.hk (J.L.L.T.); 2 Research Centre of Infection and Immunology, The University of Hong Kong, Hong Kong, China; 3 State Key Laboratory of Emerging Infectious Diseases, The University of Hong Kong, Hong Kong, China; 4 Faculty of Dentistry, The University of Hong Kong, Hong Kong, China; botelho@hku.hk; 5 Department of Pathology, The University of Hong Kong, Hong Kong, China; reginalo@pathology.hku.hk; 6 State Key Laboratory of Liver Research, The University of Hong Kong, Hong Kong, China; 7 Carol Yu Centre for Infection, The University of Hong Kong, Hong Kong, China

**Keywords:** arginine metabolism, arginase, arginine deiminase, bacterial pathogenesis, cancer therapy

## Abstract

Antibacterial resistance to infectious diseases is a significant global concern for health care organizations; along with aging populations and increasing cancer rates, it represents a great burden for government healthcare systems. Therefore, the development of therapies against bacterial infection and cancer is an important strategy for healthcare research. Pathogenic bacteria and cancer have developed a broad range of sophisticated strategies to survive or propagate inside a host and cause infection or spread disease. Bacteria can employ their own metabolism pathways to obtain nutrients from the host cells in order to survive. Similarly, cancer cells can dysregulate normal human cell metabolic pathways so that they can grow and spread. One common feature of the adaption and disruption of metabolic pathways observed in bacterial and cancer cell growth is amino acid pathways; these have recently been targeted as a novel approach to manage bacterial infections and cancer therapy. In particular, arginine metabolism has been illustrated to be important not only for bacterial pathogenesis but also for cancer therapy. Therefore, greater insights into arginine metabolism of pathogenic bacteria and cancer cells would provide possible targets for controlling of bacterial infection and cancer treatment. This review will summarize the recent progress on the relationship of arginine metabolism with bacterial pathogenesis and cancer therapy, with a particular focus on arginase and arginine deiminase pathways of arginine catabolism.

## 1. Introduction

Globally, infections and cancers are two of the leading causes of death. Bacterial infections are among the most extensively studied conditions as bacteria have established a broad range of sophisticated strategies to adapt to various environmental stresses and can resist some of the host defenses, such as chemical bactericidal components, phagocytosis (innate), and other immune (adaptive) responses [1,2]. The versatile ability of bacteria to resist host defenses allows bacteria to survive in an infection and promote their own life cycle through replication and spread in host cells [2]. With the recognition of the metabolic differences between normal cells and tumor cells, the therapeutic targeting of the cellular metabolism of cancer cells is an increasing field of research for cancer therapy [3,4]. The active deprivation of amino acids inside tumor cells, which are auxotrophic for these amino acids, has been recognized as one of the novel approaches for cancer therapy [5]. In this review, we will summarize the recent progress on the relationship of arginine metabolism with bacterial pathogenesis and cancer therapy, with a particular focus on the arginase and arginine deiminase (ADI) pathways of arginine catabolism.

## 2. Arginine Metabolism and Bacterial Pathogenesis

l-arginine is categorized as a conditionally essential amino acid in human beings. This is because it is possible to derive arginine by *de novo* biosynthesis and absorption from consumed diet [6]. In addition, l-arginine is a metabolically flexible amino acid and is metabolically interconvertible with a range of amino acids such as proline and glutamate. l-arginine is also involved in the synthesis of metabolites such as nitric oxide, creatine, polyamines, agmatine, and metabolites of the urea cycle in the cellular metabolic pathways [4,7,8].

### 2.1. Arginase Pathway and Bacterial Pathogenesis

#### 2.1.1. Arginase Pathway

**Figure 1:**
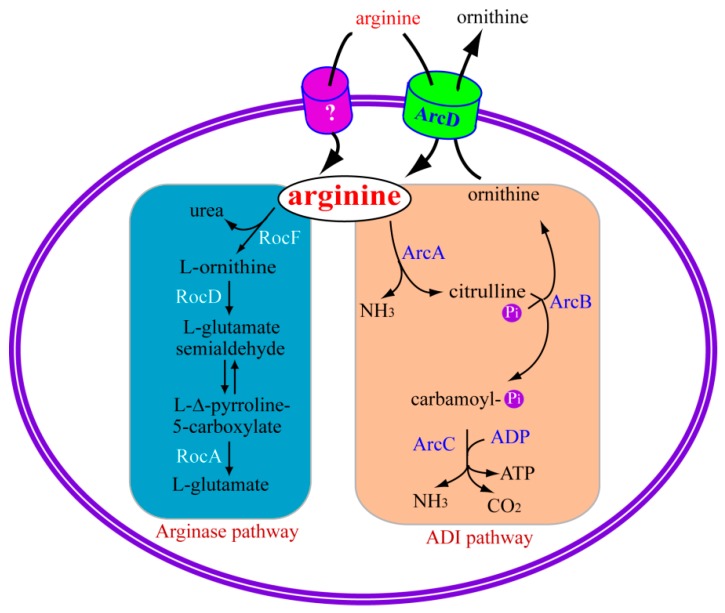
Simplified model for bacterial arginine catabolism by arginase and ADI pathways. In bacteria, arginine could be catalyzed by the arginase pathway (in blue) and/or the ADI pathway (in light salmon). For the arginase pathway, arginine is converted into urea and ornithine, which is subsequently catalyzed into glutamate. The ADI pathway catabolizes arginine to ornithine with the byproducts of ammonia, CO_2_ and ATP. The produced ornithine could be transported outside and exchange one molecule of arginine in the cell by the arginine–ornithine antiporter (ArcD) located in the bacterial membrane. Arginine may also be transported by some unknown transporters, which are shown by the question mark. RocD: ornithine aminotransferase; RocF: arginase; RocA: Δ-pyrroline-5-carboxylate dehydrogenase; ArcC: carbamate kinase; ArcA: arginine deiminase; ArcB: ornithine carbamoyltransferase; Pi: inorganic phosphate.

The arginase enzyme and its associated pathway is one arm of arginine catabolism. The arginase pathway hydrolyzes arginine to urea and ornithine, which is then hydrolyzed by ornithine aminotransferase (RocD) and Δ-pyrroline-5-carboxylate dehydrogenase (RocA) respectively, with the production of glutamate [7] (Figure 1). *Bacillus subtilis* is one of the most studied bacteria for examining the arginase pathway, and is the only pathway of arginine catabolism in *B. subtilis*, with the production of intermediaries (Figure 1) that can be broken down as a source of nitrogen [9]. In *B. subtilis*, *rocABC* [10] and *rocDEF* [11] operons and the *rocG* gene [12], are responsible for producing proteins of the arginase pathway. Arginase is encoded by gene *rocF* in *B. subtilis* and is responsible for catalyzing the first step of the arginase pathway to catabolize arginine [7]. The product from this step (ornithine) is then hydrolyzed by ornithine aminotransferase (RocD) and Δ-pyrroline-5-carboxylate dehydrogenase (RocA), respectively, with the production of glutamate (Figure 1). *rocC* and *rocE* encode arginine permeases and RocB probably function as a citrullinase [11,13]. Glutamate can be further catabolized by glutamate dehydrogenase (GDH), which is encoded by *rocG* gene, with the production of 2-ketoglutarate. Some bacteria employ this pathway to consume arginine and function as the supplier of carbon and/or nitrogen source. For example, if the bacteria also contain the urease system, the byproduct of urea from this pathway could be further catalyzed to ammonia and used as nitrogen source [7]. The genes from the arginase pathway of bacteria and their corresponding products are summarized in Table 1. In addition, their counterparts with similar catabolic function or homologue in mammalian cells (if any) are also listed.

#### 2.1.2. Regulation of Arginase Pathway

Gene expression can be regulated at different stages including transcription, post-transcription, translation, and post-translation. However, the most generally used mechanism of gene regulation in bacteria lies at the transcriptional level [19], and proper transcriptional regulation is crucial for bacteria to respond to varied environmental niches [20]. The transcriptional regulation occurs by binding to the promoter regions in induction or repression manner [19], which could be mediated by transcription factors, environmental stresses, and some other effectors.

Regulation of the arginase pathway has also been well studied in *B. subtilis*. Both *rocABC* and *rocDEF* operons, encoding enzymes for the arginase pathway, have similar promoter elements [10,11], and their transcription is driven by a specialized protein (sigma factor) encoded by the *sigL* gene, which has the same function as that of protein sigma 54 in Gram-negative bacteria [21]. In addition, the transcription of *rocABC* and *rocDEF* operons promoters is positively regulated by the regulator RocR, which is a transcriptional regulator from the protein family of NtrC/NifA, with a molecular weight of 52 kDa [7,10,13]. RocR functions by binding to enhancer-like elements (upstream activating sequences, UASs) of promoter regions of *rocABC* and *rocDEF* operons [13]. The expression of *rocR* is negatively autoregulated as the N-terminal part of RocR is an intramolecular repressor domain, and deletion of this domain makes the constitutive expression of the *roc* operons [11,13]. Furthermore, the expression of *rocABC* and *rocDEF* operons is also affected by another regulator named AhrC [13,22]. AhrC is a homologous molecule to the arginine transcriptional regulator ArgR in *Escherichia coli*, which usually binds to the promoter regions of target genes [11,13,22,23]. Similarly, it was shown that AhrC can bind to the promoter region of *rocA* in a footprinting experiment [22]. Interestingly, it was proposed in another study that AhrC should function by direct protein–protein interaction with RocR [13]. Besides, the expression of the *rocDEF* operon was also observed to be induced by the presence of arginine, ornithine, or proline in different environmental niches [11,13].

#### 2.1.3. The Relationship between the Arginase Pathway and Bacterial Pathogenesis

**Figure 2:**
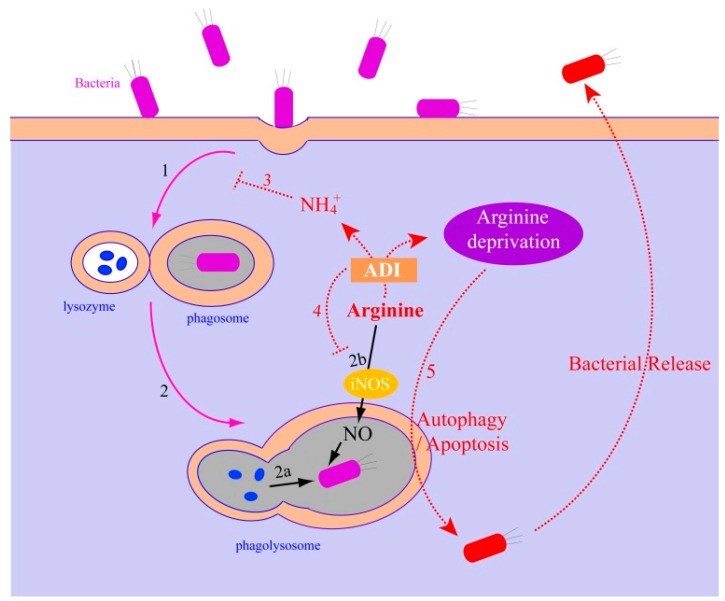
Proposed model for intracellular killing of bacteria by phagocyte and bacterial defense strategies against phagocytosis. Bacteria could be engulfed by a phagocyte into the phagosome (**1**), followed by fusion with a lysozyme to form a phagolysosome (**2**), being killed by varied strategies like pH decrease, enzymes (solid blue oval) release (**2a**), and production of antimicrobial NO by iNOS (**2b**). We propose that the bacteria containing ADI pathway genes may employ this pathway to defend these killing strategies in the following ways: firstly, the production of ammonia could probably raise the cytoplasmic pH, thereby inhibiting the formation of phagolysosome (**3**); secondly, the ADI pathway competes with iNOS for the common substrate (arginine), thereby reducing NO production (**4**); thirdly, arginine depletion would also activate the autophagy and/or apoptosis pathways, like that in cancer cells (Figure 3), to induce programmed cell death and release bacteria (**5**).

Arginine is the common substrate of arginase and inducible type 2 nitric oxide synthase (NOS2 or iNOS), which can be catabolized by arginase to ornithine and urea (Figure 1) or oxidized by iNOS to l-citrulline and nitric oxide (NO) (Figure 2) [24]. Therefore, arginase is well studied for competing with the NOS enzymes for substrate of arginine, thereby limiting NO production by different research groups [8,24]. Besides the substrate competition, arginase was also observed to inhibit iNOS expression at the translation level, thereby limiting NO production [8]. NO is a crucial element of innate immunity and is one of the valuable antimicrobials of the host’s first line of defense [25,26]. Pathogenic bacteria have developed different strategies targeting arginine for self-preservation. Similar to the arginase of mammalian cells, the arginase produced by bacteria can also compete with iNOS of host cells for the common substrate (arginine), thereby inhibiting the NO production and facilitating evasion of the host defense system [27,28]. The competition between arginase and iNOS has been reported to affect the outcome of infection of several pathogenic bacteria by modulating the NO production [29]. Among these pathogenic bacteria, one of the extensively studied examples is *Helicobacter pylori*, which releases its arginase to downregulate eukaryotic NO production so as to evade the host immune response, thereby serving as a strategy for bacterial survival [7,28]. The arginase of *H. pylori* was observed to resist acidic conditions *in vitro* and in macrophages, thereby reducing antibacterial effects [28,30]. Furthermore, arginase was employed by *H. pylori* to deplete l-arginine in macrophages, thereby limiting NO production and prolonging bacterial survival. Deletion of the *rocF* gene that is responsible for arginase production significantly increased NO production and thereby decreased the survival of the *rocF* gene mutant strain in macrophages [28,31]. However, the survival of *rocF* gene deleted *H. pylori* was not affected in the macrophages from iNOS^−/−^ mice, indicating the survival decrease in arginase-deficient *H. pylori* was NO-dependent. In addition, arginase from *H. pylori* also represses the expression of the TCR ζ (CD3ζ) chain, thereby restraining the function of the host T cells, which can also contribute to bacterial survival during *Helicobacter* infection [32].

*H. pylori* were found to upregulate arginase (arginase II) in RAW 264.7 macrophage cell lines, in a mouse model, and in human gastritis tissues. This upregulation was found to induce apoptosis. The action of arginase on arginine can produce an intermediary spermine, with the help of ornithine decarboxylase and spermine synthase. This is used by *H. pylori* to repress the expression of pro-inflammatory cytokines and iNOS, thereby preventing the antimicrobial effects of NO and the immune response in stimulated macrophages [33]. Of note, it was shown that host arginase (arginase II), which was upregulated to reduce NO production, can increase bacterial survival in *Salmonella*-infected macrophages [34]. Arginase has also been shown to be involved in bacterial infection caused by *Mycobacterium tuberculosis* [35,36], parasitic infection caused by *Trypanosoma cruzi* [37], and fungal infection caused by *Candida albicans* [38], implying the critical role of arginase for other infections.

### 2.2. Arginine Deiminase Pathway and Bacterial Pathogenesis

#### 2.2.1. Arginine Deiminase Pathway

Arginine metabolism via the ADI pathway is well established to be broadly present in a number of microorganisms and enables them to adapt to hostile environmental niches and host defenses [14,15,16,17,39,40]. As one of the most important arginine catabolism pathways, the ADI pathway is a multi-enzyme pathway encoded by *arc* operons genes named *arcA*, *arcB*, and *arcC* [15,39], which hydrolyze arginine to ornithine, with the byproducts of ammonia, CO_2_ and ATP. Generally, arginine is first catabolized to citrulline by *arcA*-encoded ADI, which is further hydrolyzed to ornithine and carbamoyl phosphate via an *arcB*-encoded catabolic ornithine carbamoyltransferase (OTC). Finally, a phosphate is relocated from carbamoyl phosphate to adenosine diphosphate (ADP), by an *arcC*-encoded enzyme named carbamate kinase (CK), with the production of ammonia, carbon dioxide, and ATP. The production of ammonia by the ADI pathway could further produce NH4^+^ and thus increase the cytoplasmic pH, thereby protecting the bacteria from being killed by the hostile acidic conditions [15]. Furthermore, the production of ATP can provide energy for bacteria to pump cytoplasmic protons and maintain cytosolic pH homeostasis [15]. Another element of this pathway, an arginine–ornithine antiporter (ArcD) encoding by *arcD*, could transport ornithine out and exchange a molecule of arginine into the cell (Figure 1) [14,15,18]. The genes from the ADI pathway of bacteria and corresponding products are summarized in Table 1. Their counterparts with similar catabolic function or homologue in mammalian cells (if any) are also listed.

#### 2.2.2. Regulation of Arginine Deiminase Pathway

As stated in Section 2.1.2, the most extensively studied regulatory mechanism of the ADI pathway also lies at the transcriptional level, which is mediated by the transcriptional regulator ArgR [9,15,17]. ArgR could be derived from two different protein families: ArgR/AhrC [41,42,43] and AraC/XylS [9,44,45]. Irrespective of the family origin, ArgR proteins generally elicit their regulatory role by binding to the ARG operator (ARG box) located in the *arc* operon promoter [46,47], via a conserved helix-turn-helix motif in the C-terminal of ArgR [48]. Generally, ArgR regulator activates the expression of ADI pathway genes in their individual bacteria [15,17,45,46,49]; the only exception is the ArgR of *Laribacter hongkongensis*, a novel beta proteobacterium associated with gastroenteritis [50,51], which has been illustrated to have dual regulatory functions on two adjacent *arc* operons within *L. hongkongensis* [52].

Besides the transcriptional regulation by ArgR, ADI pathway genes are also affected by a variety of environmental stresses. Firstly, the expression of ADI pathway genes are established to be activated by anaerobicity in different bacteria [53], such as *Pseudomonas aeruginosa, Bacillus licheniformis*, and *Streptococcus suis*, mediated by transcriptional regulators from the protein family of Crp/Fnr [54,55,56]. In enteric anaerobic bacteria, regulators from the Crp/Fnr family recognize conserved Crp/Fnr DNA-binding sites located in the *arc* operon promoter [57,58], enabling them to function as a transcriptional activator. Furthermore, carbon catabolite repression (CCR) has also been demonstrated on the expression of ADI pathway genes in various bacteria [40,56,59,60]. In these bacteria, the expression of *arc* operon genes is repressed by glucose and the repression is usually mediated by a catabolite control protein A (CcpA) or ArcR, the transcriptional regulators belonging to the Crp/Fnr family [40,56,59], by the binding of regulatory proteins to the *cis*-acting catabolite response elements (*cre*) located in the promoter regions [61]. Moreover, bacteria have to survive and inhabit diverse environmental niches or hosts, so they have complex regulatory mechanisms to sense and respond to different environmental stresses; for example, it has been shown that the expression of the ADI pathway of *S. suis* and *L. hongkongensis* occurs at varied temperatures, allowing better adaptation and survival in different environments [52,62].

#### 2.2.3. The Relationship between the ADI Pathway and Bacterial Pathogenesis

Due to the great importance of arginine metabolic functions, the ADI pathway has been demonstrated to be necessary for bacterial survival under acidic conditions and important for bacterial virulence in varied bacteria [14,15,16,17,56,63,64]. The ammonia produced by the ADI pathway can neutralize the cytoplasmic pH and protect the cell from the potentially lethal effects of acidic extracellular environments [15,16,65]. Furthermore, ADI was demonstrated to be required for *Streptococcus pyogenes* to invade and survive inside epithelial cells, and necessary for the intracellular survival of *L. hongkongensis* in macrophage cell lines [14,16]. Additionally, mutation of ADI gene has been shown to reduce the survival of *Listeria monocytogenes* in the spleen of a mouse infection model [15]. Importantly, the ADI of *Salmonella enterica* serovar Typhimurium was recently established as a virulence factor [17]. It was demonstrated that the expression of the ADI pathway genes of *S.* Typhimurium was elevated after infecting macrophages cell lines, and disruption of ADI gene significantly reduced the bacterial replication in murine macrophages and attenuated the bacterial virulence in a mouse model, when compared to the wild-type strain [17].

Although studied extensively, the precise mechanism by which the ADI pathway enhances intracellular survival of bacteria and even their replication within macrophages is still unclear [16]. However, it is believed that there is some connection between phagocytosis and the ADI pathway [16,17,66]. As an important defense strategy in innate immunity, phagocytosis has always been employed by our immune system to initiate the innate immune response and destroy infectious microorganisms [67]. In the process of phagocytosis, bacteria are enclosed in the phagosome [68,69], which would undergo maturation and fuse with the lysosome, to form the phagolysosome [70], where the pH drops as low as 4.4–4.7 [71,72,73,74]. Since the ADI pathway has the property of increasing the cytoplasmic pH, it would be employed to protect bacteria from the potentially lethal effect of acidic extracellular environments [15]. The ADI pathway can also protect bacteria from being killed in the phagolysosome, via arresting the pH decrease (Figure 2), thereby preventing the phagosome and lysosome from fusing. Bacteria ingested by phagocytes can be degraded in phagolysosomes in different ways, including proteolytic enzymes like lysozymes, cationic proteins, and production of antimicrobial products [17,75]. Of these antimicrobials, NO is synthesized from arginine by iNOS, and the availability of arginine is one of the rate-limiting factors for intracellular NO production [76,77,78]. As the ADI pathway consumes arginine as a substrate, it appears that bacteria can use the ADI pathway to exhaust arginine in the host cell. This will reduce NO production, avoid NO-mediated killing in the phagocytes, and increase bacterial survival (Figure 2). However, a study on *S.* Typhimurium excluded this possibility as the wild-type and ADI mutant strains have similar levels of NO production after infection in macrophages [17]. More studies are needed to investigate other mechanisms in further bacteria [2]. Remarkably, recombinant ADI (rADI) protein was employed as the neuroprotective protein and was capable of blocking iNOS-induced NO production in neuronal cells as the neuroprotective protein [79]; whether pathogenic bacteria would employ this strategy to escape from phagocytosis killing warrants further investigation. There could also be many other possibilities. For example, one proposed strategy is that the production of ATP in the process of ADI pathway enables bacteria to provide energy for F_1_F_O_-ATPase to pump cytoplasmic protons and so maintain cytosolic pH homeostasis under acidic environments. Similar strategies have been employed by many pathogenic bacteria to survive within acidified phagosomes [80,81,82,83].

## 3. Arginine Metabolism and Cancer Therapy

Disorder of cellular metabolism has been accepted as one of the critical marks for cancer production and expansion [3,4]; this has stimulated research on cancer metabolism and both the basic science and clinical treatment of cancer [3,84]. The metabolic differences between normal cells and tumor cells have provided opportunities for developing novel approaches for the diagnosis and treatment of cancer with higher specificity and lower toxicity than conventional cancer therapies like radiation and chemotherapy [4,85,86]. Of note, it is becoming clear that amino acid metabolic pathways could be chemotherapeutic targets for cancer therapy, as cancer cells need abnormal quantities of varied amino acids for their distinct metabolism to maintain high proliferative rates and resist some cell death signals [87]. Consequently, the deprivation of amino acids needed by cancer cells to survive has been recognized in the field of cancer therapy for a long time [5], especially for some cancers that are auxotrophic for specific non-essential amino acids [4,6].

### 3.1. Arginine Deprivation and Cancer Therapy

**Figure 3:**
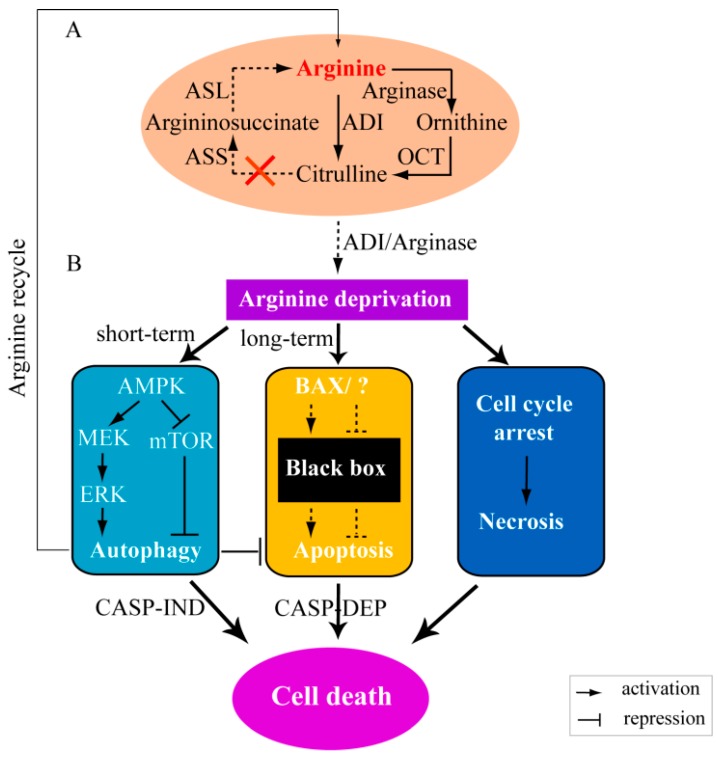
Schematic representation of argininosuccinate synthetase (ASS)-negative cell death induced by arginine deprivation. In ASS-negative cells, arginine cannot synthesize so arginine depletion by ADI or arginase would induce a quick response of cell autophagy by the mammalian Target of Rapamycin (mTOR) or MEK-ERK pathway. Autophagy could recycle limited arginine and prevent apoptosis as a survival response in the short term. Instead, in long-term arginine deprivation, autophagy would contribute to caspase-independent (CASP-IND) cell death and caspase dependent (CASP-DEP) apoptosis could also happen. The dashed lines (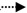 and 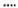) means the reactions are dependent on the availability of enzymes (panel **A**) or the reactions have not yet confirmed by experiments (panel **B**). MEK: mitogen-activated protein kinase, also known as extracellular signal-regulated kinase kinase; ERK: extracellular signal-regulated kinase.

The relationship between arginine and cancer has been recognized for many years [88]. Generally, *de novo* biosynthesis of arginine is from the precursor argininosuccinate, which in turn is produced from citrulline; this is facilitated by the enzymes argininosuccinate lyase (ASL) and ASS, respectively (Figure 3) [4,89]. ASS is the rate-limiting biosynthetic enzyme for intracellular arginine synthesis in different cells [86,89]; however, in some tumor cells it is reduced or even absent, like hepatocellular carcinoma (HCC), mesotheliomas, renal cell carcinoma, prostate cancers, and the majority of melanoma [90,91,92] (Table 2). These ASS-negative tumor cells are auxotrophic (dependent on uptake of extracellular arginine) for arginine and thus are very sensitive to arginine deprivation [4,85,89]. The clinical relevance of arginine metabolism in cancers was illustrated by the association of reduced ASS and more aggressive clinical behavior in pancreatic cancer [93]. In addition, evaluation of ASS1 levels in clinical samples of acute myeloid leukemia is deemed a promising direction for identifying patients who would be more sensitive to arginine deprivation therapy [94]. Consequently, arginine deprivation by arginine-degrading enzymes has been used as a therapy for selective tumor cell death while not harming normal cells [89]. However, only ADI and arginase are the commonly investigated arginine-degrading enzymes in basic and clinical research. This is because the other arginine-degrading enzymes have limitations, such as low affinity with arginine, higher optimal pH, and poor stability [6,85,88].

Arginase is responsible for hydrolyzing arginine to ornithine, which in turn can be converted to citrulline by Ornithine carbamoyl transferase (OCT), thereby depleting the arginine in the arginine auxotrophic cancer cells (Figure 1 and Figure 3). There are two subtypes of arginase in humans (arginase I and arginase II). Arginase I is mainly found in the liver and is considered the more efficient subtype and used more commonly in research [6]. Compared with the arginase of bacteria, the arginase from human sources has the advantage of low immunogenicity for use *in vivo*. However, it has been observed to have a low affinity with arginine *in vitro* [4] and requires a higher pH than physiological conditions for optimal activity (up to 9.5) [116], which limits its clinical effectiveness in cancer therapy. To overcome these, recombinant human arginase (rhArg-PEG) has been produced by linking polyethylene glycol (PEG) to the arginase, which increases its affinity with arginine and improves the half-life *in vivo* [117]. In addition, the replacement of Mn^2+^ with Co^2+^ in the active site of arginase can reduce the optimal pH of recombinant human arginase (rhArg1) to as low as 7.5 and this metal ion replacement also enhances rhArg1 cytotoxicity in hepatoma cancer cell lines [118]. Recently, other recombinant human arginase (rhArg1-Fc) has been produced by ligating Fc fragment of human IgG1 to arginase I; this greatly increases the half-life, inhibits cell proliferation, and impairs cellular migration of different tumor cell lines both *in vitro* and *in vivo* [6,119].

Arginine deiminase is another well-studied arginine-depriving enzyme and this degrades arginine to citrulline, which can be recycled back to arginine by ASS and ASL in many cell types but not in ASS-negative cancer cells (Figure 3) [88,120]. The application of ADI for anti-tumor therapy in cancer cell lines and animal models was demonstrated over two decades ago [121,122]. As ADI is not produced in mammals, ADI protein for research is most commonly derived from *Mycoplasma* *arginini* [6]. However, because of its short half-life and high antigenicity, ADI is linked with PEG (ADI-PEG20) and has been used for research and clinical use [4,123]. This has been observed to have significantly reduced antigenicity, increased half-life [123], and high affinity with arginine (about 300-fold that of arginase under physiological pH) [85,101,124]. Because of its efficacy, it has been widely used in clinical trials for anti-cancer treatment [105,106,125]. Of note, a recent study of neuroblastoma cells has shown ADI-PEG20 to have potential for treating iNOS-mediated neurodegenerative diseases [126]. Importantly, it was reported that ADI-PEG20 treatment in mesothelioma patients can produce anti-ADI-PEG20 neutralizing antibodies by the fourth week, thereby resisting drug usage; further drug optimization is still needed to reduce ADI treatment’s immunogenicity, replace it with human recombinant arginase I, and combine with various drugs [3,127,128].

### 3.2. Molecular Mechanisms of Arginine Depletion for Cancer Therapy

#### 3.2.1. Arginine Deprivation Induces Autophagy in ASS-negative Cells

Arginine is an essential amino acid in ASS-negative tumor cells (Table 2) as these arginine auxotrophic cells lack the ASS enzyme (Figure 3) responsible for generating arginine from citrulline and therefore have to uptake extracellular arginine. It has been demonstrated that arginine depletion in these cells causes nutritional deprivation and consequently induces autophagy, so that application of arginine deprivation enzymes has been undertaken for cancer therapy [5,85,101]. Autophagy is a highly regulated cellular pathway in which the constituents are sequestrated into double-membrane compartments (known as autophagosomes) and fused with lysosomes for degradation [129,130,131]. This process is the principal catabolic pathway to nutrient starvation [86,89,129,132]. Autophagy can be activated by a range of pathways, one of which is the mTOR pathway.

During nutritional deprivation, the mTOR pathway is inhibited; this in turn inactivates some of the non-essential energy consuming intracellular processes to preserve cell viability. This protective process is an extensively investigated pathway for activating autophagy and can be triggered by arginine depletion [5,85,101]. ADI treatment can cause arginine deprivation of tumor cells, which in turn activates AMP-activated protein kinase (AMPK) [5,101]. This molecule is recognized to inhibit mTOR activity via energy/nutrient sensing [109,133,134] (Figure 3) and so induce autophagy. Furthermore, the activated AMPK induced by ADI treatment could also activate the MEK-ERK pathway, which has been reported to be involved in autophagy by regulating Beclin 1 via noncanonical pathway [135], suggesting that the MEK-ERK signaling pathway was also activated to induce autophagy by arginine depletion [85,101]. Together, arginine deprivation by ADI treatment in ASS-negative cancer cells activates AMPK, which further represses the mTOR activation and stimulates the MEK-ERK pathway, thereby inducing autophagy activation. This is a survival strategy for cancer cells as the limited arginine can be recycled and provide protection for other cells [85].

Apart from autophagy, arginine metabolism was demonstrated to modulate chemosensitivity of cancer cells. In particular, ADI reinforced the effects of gemcitabine on pancreatic cancer cells [93]. This was achieved through regulation of cell cycle progression, the caspase system, as well as the NF-κB pathway.

#### 3.2.2. Arginine Deprivation Prompts Cell Death in ASS-negative Cells

Arginine deprivation has also been reported to induce apoptosis and cause cell death in ASS-negative tumor cell lines, including human lymphoblastic cell lines, mesothelioma cells, melanoma cell lines, prostate cancer cell lines, and breast cancer [5,89,95,101,113,136,137]. Although the signaling pathway responsible for apoptosis is still not clear, it is recognized that the apoptosis induced by arginine deprivation could be activated via caspase-dependent and/or independent pathways [85,102]. Szlosarek *et al.* showed that arginine depletion in ASS-negative mesothelioma cells induced apoptosis via Bcl-2-associated X protein (BAX) activation and mitochondrial inner membrane depolarization [113]. Importantly, excessive autophagy could be cytotoxic and also cause cell death (program type II or caspase-independent cell death) [86,89,138]. Kim *et al.* observed that autophagy induced by arginine depletion in ASS-negative cells could lead to apoptotic cell death in a caspase-independent manner at four days after ADI treatment [101]. In ASS1-deficient breast cancer cells, prolonged autophagy activated by ADI treatment impaired mitochondrial bioenergetics and integrity by inducing mitochondrial oxidative stress, and finally caused cell death, indicating the relationship of the cytotoxic autophagy arisen by mitochondrial dysfunction with cell death [89]. Furthermore, Changou *et al.* observed that mitochondrial damage played a central role in new atypical cell death induced by arginine starvation in the late phase of autophagy. This can lead to reactive oxygen species generation, DNA leakage, chromatin autophagy, and finally cell death [86]. Remarkably, the recombinant human arginase (rhArg) treatment restrains the proliferation of mammalian melanoma *in vitro* and *in vivo* and causes cell death induced by apoptosis [96]. The reason why rhArg affects cell proliferation is that the rhArg can induce the cell cycle arrest at both of G2/M and S phase. Similarly to arginine depletion caused by ADI treatment, the cell cycle arrest caused by rhArg also upregulates the caspase expression, thereby inducing apoptosis [96].

The processes of autophagy and apoptosis are related in ASS-negative cells. In the early stage of nutrient deficiency, autophagy will be triggered to recycle limited arginine and preserve the tumor cell and this will have an inhibitory effect on apoptosis. However, when the cells undergo arginine depletion for a longer time and autophagy cannot provide arginine anymore, the cells will undergo caspase-independent apoptosis. Meanwhile, caspase-dependent apoptosis could also be activated by ADI treatment via some other signaling pathways, for example BAX activation (Figure 3). In addition to the arginine deprivation-caused cell death by autophagy and apoptosis pathways, cell death induced by necrosis was also observed in acute myeloid leukemia (AML) treated with pegylated (PEG) recombinant human arginase (BCT-100) [110]. BCT-100 treatment in AML was observed to reduce the arginine concentration (*in vitro* and intracellular) and AML blasts. Furthermore, the arginine depletion of BCT-100 treatment significantly arrested AML proliferation and the cell cycle, resulting in cell death of necrosis, including cell membrane permeabilization and organelle enlargement [110]. Interestingly, the cell cycle arrest in AML did not induce cell apoptosis, autophagy, and rapid production of reactive oxygen species.

## 4. Conclusions

Our recent findings on the biochemical pathways of arginine metabolism and their regulation in bacteria and cancer cells have not only improved our knowledge of the pathogenesis of bacterial pathogens and cancer metabolism, but may also result in specific anti-bacterial and anti-cancer therapies. Further work in these areas is warranted to improve our armory of strategies against these two common groups of diseases affecting human health.

## Figures and Tables

**Table 1:** The genes from arginase and ADI pathway of bacteria and counterparts in mammalian cells with homologue or similar function.

Pathway (Genes)	Products	Counterparts in Mammalian Cells (with Similar Function or Homologue)	Source/Reference
The arginase pathway			
*rocA*	Pyrroline-5-carboxylate dehydrogenase	Pyrroline-5-carboxylate dehydrogenase	[10]
*rocB*	Probable citrullinase	-	[13]
*rocC*	Arginine permease	Arginine permease-like	[11,13]
*rocD*	Ornithine aminotransferase (OAT)	Ornithine aminotransferase	[11]
*rocE*	Arginine permease	Arginine permease-like	[11,13]
*rocF*	Arginase	Arginase I and II	[11]
The ADI pathway			
*arcA*	Arginine deiminase	Nitric oxide synthase (NOS)	[14,15,16,17,18]
*arcB*	Ornithine carbamoyltransferase	Ornithine carbamoyltransferase	[14,18]
*arcC*	Carbamate kinase	Carbamate kinase-like	[15,16]
*arcD*	Arginine-ornithine antiporter	-	[16,17,18]

**Table 2:** Common cancer cells with reduced or absent ASS production.

Cancer Cell Types	Source or Reference
Melanoma	[5,85,90,93,95,96,97,98,99,100]
Breast cancer cells	[89]
Prostate cancer cells	[4,86,90,101,102]
Lymphoma	[89,103,104]
Hepatocellular carcinoma (HCC)	[5,90,93,98,100,105,106,107]
Pancreatic cancer cells	[91,93,108]
Leukemia	[94,109,110,111]
Glioma	[89,112]
Mesothelioma cell lines	[5,113]
Renal cell carcinoma	[4,93,114]
Lung cancer	[93,115]
